# Differential involvement of caspase-6 in amyloid-β-induced fragmentation of lamin A and B

**DOI:** 10.1016/j.bbrep.2020.100839

**Published:** 2020-10-26

**Authors:** Md Imamul Islam, Md Selim Hossain, Il-Seon Park

**Affiliations:** a Department of Medical Sciences, Republic of Korea; b Department of Cellular and Molecular Medicine, Chosun University, Gwanju, 501-759, Republic of Korea

**Keywords:** Amyloid β, Alzheimer's disease, Lamin, Nuclear scaffold protease, Caspase-6, Phosphorylation, Aβ42, 42-amino-acid amyloid β, AP, alkaline phosphatase, AD, Alzheimer's disease, ANU, nuclei isolated from Aβ42-treated cells, HNU, nuclei isolated from healthy cells, NSP, nuclear scaffold protease, STS, staurosporine

## Abstract

Amyloid-β (Aβ), a peptide implicated in Alzheimer's disease, was shown to cause specific fragmentation of lamin proteins, which was mediated by an unidentified protease named nuclear scaffold protease (NSP) independently of caspase-6. Because caspase-6 is responsible for the fragmentation process in many other damage-induced apoptosis, here we further investigated possible involvement of caspase-6 in Aβ-induced lamin fragmentation under various conditions. We found that lamin A fragment generated by NSP (named fragment b) disappeared in cells incubated with Aβ42 for prolonged periods and this product was preserved by a caspase-6 inhibitor. Furthermore, caspase-6 could remove fragment b in nuclei isolated from Aβ42-treated cells (ANU). Lamin B in ANU was fragmented by caspase-6 only after treatment with an alkaline phosphatase. The caspase-mediated fragmentation of lamin B was also achieved with nuclei isolated from cells incubated with Aβ42 plus a Cdk5 inhibitor. The results indicate that Aβ42 induces NSP-mediated fragmentation of lamin A and the following removal process of fragment b by caspase-6 and an Aβ-induced phosphorylation prevents the fragmentation of lamin B by caspase-6. The pathway leading to lamin protein fragmentation in this investigation appears to be specific for Aβ and thus the data will provide novel insights into the toxicity of the peptide.

## Highlights

• Aβ42 induces nuclear scaffold protease (NSP)-mediated fragmentation of lamin A.
• The produced fragment of lamin A is subsequently removed by caspase-6.
• Aβ42 also induced NSP-mediated lamin B fragmentation.
• Caspase-6-mediated fragmentation of lamin B protein is absent.
• The absence appears to be due to phosphorylation of lamin B.

## 1 Introduction

Amyloid-β (Aβ) peptide is a major component of the extracellular senile plaques implicated in the neurodegenerative Alzheimer's disease (AD) [1]. The conformational change of the 36- to 43-amino-acid long Aβ peptide to β-sheet-rich intermediate structures leads to the formation of soluble multimeric Aβ species such as oligomers and protofibrils that show superior toxicity to insoluble fibril deposits formed by the subsequent fibrillogenic process [2,3]. Aβ peptide induces cellular events that could lead to cell death. One such event that we previously reported is fragmentation of lamin proteins [4]. Polymerized lamin proteins are the major structural components of the nuclear scaffold. They are essential for nuclear functions such as DNA replication, transcription, and repair [5]. Thus, the nuclear lamina deformation and fragmentation observed in Aβ-treated cells could result in cell death.

Fragmentation of lamin proteins could be mediated by at least two enzymes [4]. One of these enzymes is caspase-6, which is involved in the lamin fragmentation in apoptosis induced by various types of damages [4,6,7]. However, fragmentation of lamin A in Aβ-treated cells was apparently not mediated by caspase-6, because a ~46 kDa N-terminal fragment of lamin A (fragment b, see Fig. 2G) was detected instead of a ~28 kDa fragment (fragment a) that was expected to be produced by the caspase [4]. Similarly, a ~46 kDa C-terminal lamin B fragment (fragment c) that was expected upon fragmentation by caspase-6 was not detected in Aβ-treated cells and instead, ~21 kDa fragment d was seen [4]. Thus, we proposed that another enzyme named nuclear scaffold protease (NSP) which was not yet identified could be responsible for Aβ-induced lamin fragmentation [4,8].

In the current study, we explored several hypotheses to understand the absence of caspase-6-mediated lamin fragmentation and ultimately to identify pathways leading to fragmentation of lamin proteins in Aβ-treated cells. Evidences provided here indicate that the caspase-6-mediated fragmentation of lamin protein A could occur only after production of fragment b with activation of the caspase and an inhibitory phosphorylation of lamin B prevented caspase-6-mediated fragmentation of the protein.

## 2 Materials and methods

### 2.1 Cell culture and caspase activity assay

Human epithelial HeLa cells and human neuroblastoma SHSY5Y were cultured cells as described previously [4,9]. Treatment was performed according to the mentioned concentration of oligomeric Aβ42 and time point in serum-free cell culture medium. Caspase-6 activity was measured as before [4,9] using the synthetic Ac-VEID-aminomethylcoumarin (AMC) and inhibited by Ac-VEID-CHO (A.G. Scientific Inc., San Diego, USA).

### 2.2 Preparation of Aβ42 and caspase-6

Aβ42 was purified as described previously [10]. The peptide aliquots were stored at −20 °C until use. Aβ42 oligomers were prepared according to previously published method [11]. Human caspase-6 was purified from BL21 pLys *Escherichia coli* strain conveying the gene inserted into pET28b vectors (Novagen, USA) at an N-terminal histidine tag according the previous study [12].

### 2.3 Western blotting

The immunoblot assay was performed as reported before [4,9]. Anti-lamin-A/C, anti-lamin B, and anti-β-actin antibodies were from Santa Cruz Biotechnology (California, USA). Anti-caspase-6 antibody that could detect the small domain (p11) of caspase-6 were from BD Pharmingen (CA, USA) (named as Ab1). Anti-caspase-6 antibody recognizing the large domain of the enzyme was from Abfrontier (Seoul, Korea) (named as Ab2).

### 2.4 Cell-free experiments with isolated nuclei

The nuclei prepared as described before [4] were resuspended in the resuspension buffer without Triton X-100. For the cell-free assay, isolated nuclei were incubated at 30 °C in a reaction buffer solution containing 20 mM HEPES-KOH (pH 7.5), 0.1 mM EDTA, and 1 mM DTT with the indicated chemicals or proteins. After the reaction, nuclei were lysed by adding 2 × SDS-PAGE loading buffer, separated on 10% SDS-PAGE, and immunoblotted for target proteins. (Alkaline phosphatase (AP) and roscovitine were from Santa Cruz (California, USA) and Calbiochem (California, USA), respectively.

## 3 Results

### 3.1 Probing activation conditions of caspase-6 by Aβ42

**Fig. 1:**
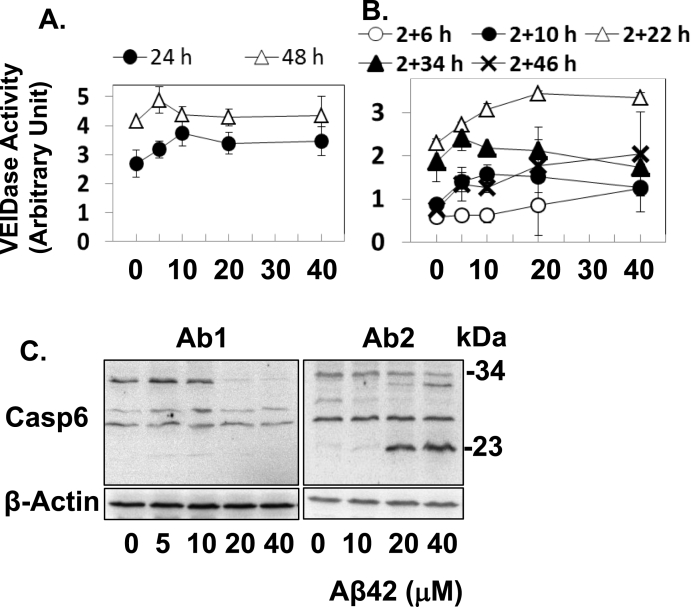
Double treatment but not single treatment with Aβ42 oligomeric species induces caspase-6 activity. (A,B) HeLa cells (2 × 10^4^/100 μl of culture media) were treated with oligomerized Aβ42 at the indicated concentrations and time. Caspase-6 activity was measured using 50 μM ac-VEID-AMC. The results are expressed as mean ± standard deviation of 3 independent experiments. (C) HeLa cells (8 × 10^5^) were treated with the indicated concentration of Aβ42 for 2+22 h, total cell lysate was prepared and processing of caspase-6 cleavage was monitored by immunoblot analysis using mouse anti-human caspase-6 monoclonal antibody (Ab1) and rabbit anti-caspase-6 polyclonal antibody (Ab2). β-Actin was used as a loading control. Relative molecular weights (in kDa) are indicated on the right. The result is representative of at least 3 equivalent experiments.

In the current study, we hypothesized that caspase-6-mediated lamin fragmentation could occur in Aβ-treated cells if the enzyme is activated enough to induce the process [[13], [14], [15], [16]]. This is because the absence of the caspase-6-mediated fragmentation in the previous study [4] could result from the low level of activation of caspases including caspase-6 which was probably due to incubation of cells with Aβ42 for short period of times (<12 h). Thus, the hypothesis was tested by examining lamin fragmentation in cells administered by Aβ42 for prolonged periods of time. Furthermore, concentrations of Aβ42 and treatment methods were determined for potent activation of caspase-6 and the fragmentation were examined under the conditions. Throughout the current study, oligomeric preparation of Aβ42 was used unless otherwise indicated, because the oligomeric Aβ species induced apoptosis better than monomeric Aβ and fibrils [3,17]. The “single” treatment of cells with Aβ42 for up to 48 h resulted in certain levels of caspase-6 activation that was evaluated by a synthetic substrate Ac-VEID-AMC (Fig. 1A). However, it was questionable if the activity was derived solely from Aβ42 treatment, because there was a weak dependency of the activity on Aβ42 concentration.

**Fig. 2:**
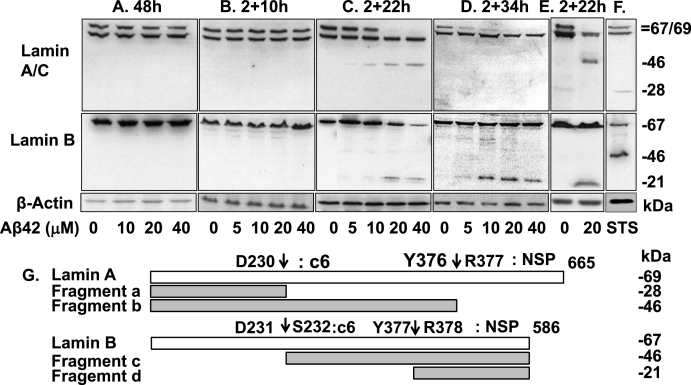
Fragmentation of lamin A and B induced by Aβ42. (A–D) HeLa cells (8 × 10^5^) were treated as indicated. (E) SHSY5Y cells were (8 × 10^5^) treated with 20 μM of oligomeric Aβ42 for 2+22 h. (F) HeLa cells were treated with 0.5 μM of STS for 6 h. Total cell lysate was prepared from each sample and was analyzed for the fragmentation of lamin A/C and B by immunoblotting (antibody for Lamin A/C was raised against the N-terminus of human Lamin A/C and that for Lamin B was raised against the C-terminus of human Lamin B1). β-Actin was used as a loading control. Relative molecular weights (in kDa) are indicated on the right. The result is a representative of at least 3 equivalent experiments. (G) Schematic drawings of lamin A and B cleavage sites by caspase-6 and NSP. The predicted sizes of the cleavage fragments are indicated on the right. The fragments were indicated by a, b, c, and d, respectively.

The Aβ42-dose dependency of caspase-6 activity was observed rather prominently in cells treated twice with Aβ42 peptide (Fig. 1B, also [4]). Among the “double” treatment conditions, the 2 h pretreatment and subsequent 22 h (2+22 h) treatment with 20 μM Aβ42 induced the most potent activation (Fig. 1B). We further probed the processing of caspase-6 using western blot analysis. Ab1 antibody which detects the small subunit of processed caspase-6 (p11) was initially used in the analysis. Although it was difficult to detect p11, the decrease in the ~34 kDa procaspase-6 was clearly visible in the 2+22 h samples with a high dose of Aβ42 (20 and 40 μM) (the left panel of Fig. 1C). The same western blot analysis was performed with another anti-caspase-6 antibody, Ab2 that recognizes the large subunit of the enzyme. Proteins of ~23 kDa corresponding to the large subunit of caspase-6 were clearly seen in the same samples as above (the right panel of Fig. 1C). Altogether, these data indicated that caspase-6 is activated in the 2+22 h samples treated with Aβ42 at 10–40 μM. The 2+22 h or longer incubated samples were employed throughout the current study unless otherwise indicated, because of the prominent caspase-6 activation.

### 3.2 Fragmentation of lamin A and B induced by Aβ42 under various conditions

**Fig. 3:**
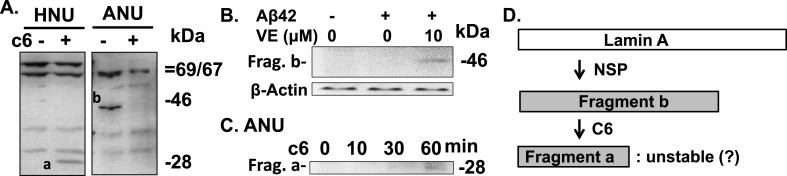
Fragmentation of lamin A by caspase-6. (A and C) Nuclei were isolated from buffer- (HNU) and Aβ42-treated cells (ANU, also see RESULTS). The nuclei were incubated with and without 50 ng of purified recombinant caspase-6 for 1.5 h (A) or indicated time (C) at 30 °C. (B) Cells were treated as described in Fig. 2D with and without ac-VEID-CHO (VE). Fragmentation of lamin A was probed by western blot analysis in all experiments. β-Actin was used as a loading control in B. Relative molecular weights (in kDa) are indicated on the right. a, b and c6 are fragment a, b and caspase-6, respectively. Data are representative of 3 independent experiments. (D) Schematic drawing of lamin A fragmentation process.

We next probed the processing of lamin A/C and B in cells treated with Aβ42. While no fragmentation of lamin proteins was observed in cells treated with Aβ42 for 48 h or 2+10 h (Fig. 2A and B), fragmentation was detected in 2+22 h and 2+34 h samples (Fig. 2C and D). The detected ~46 kDa fragment b (see Fig. 2G) and ~21 kDa fragment d, known to be produced by NSP [4,8], are not products generated by caspase-6, which is expected to produce ~28 kDa fragment a and ~46 kDa fragment c, respectively (Fig. 2G). Thus, it seems that caspase-6 was not functionally active for the fragmentation of lamin proteins in Aβ42-treated cells, although the enzyme was catalytically active as shown in Fig. 1. We also tested the lamin fragmentation in human neuroblastoma SHSY5Y cells, which showed a similar pattern of fragmentation (Fig. 2E). In the following experiments, HeLa cells were mainly used, because they showed higher caspase activity than SHSY5Y cells which showed low activity of the enzyme and high level of cell death for the following treatment and experimental procedures (data not shown). Notably, the fragment b disappeared in 2+34 h samples (Fig. 2D). We will discuss this below (see Fig. 3A). For comparison, the caspase-6-dependent fragmentation of lamin proteins was shown in cells treated with staurosporine (STS) (Fig. 2F). Lamin C will not be discussed, because it is not a substrate of NSP or caspase-6 [4].

### 3.3 Fragmentation of lamin A by caspase-6 in isolated nuclei and cells

Following hypothesis for the absence of caspase-6-mediated lamin fragmentation shown in Fig. 2 was that the lamin proteins of Aβ-treated cells might be somehow different from those of the healthy cells as a substrate of caspase-6. To test the hypothesis we compared the lamin cleavage by purified caspase-6 in nuclei isolated from healthy (HNU) and Aβ42-treated (ANU, we mainly used the 2+22 h sample with 20 μM of the peptide in the current and following cell-free experiments) cells. The rationale of using this experimental condition was to ensure the effect of exogenous caspase-6 on Aβ induced NSP mediated cleaved products of lamin proteins (Fig. 2C).

A lamin fragment corresponding to fragment a which is an N-terminal part of lamin A (Fig. 2G) was generated in HNU treated with caspase-6 (Fig. 3A, HNU panel) as expected in the western blot analysis with the antibodies used for the current investigation (see Fig. 2G). Although the absence of lamin A in ANU (Fig. 3A) precluded the direct test of the effect of caspase-6 on the fragmentation of the protein in the nuclei (Fig. 3A, ANU panel), fragment b can be a substrate, because it has a caspase-6 cleavage site (see Fig. 2G). The fragment was removed by caspase-6 (Fig. 3A, right lane of ANU panel), implying that the fragment b can be a substrate of the caspase as suggested.

We think that the ANU result above (Fig. 3A) is compatible with those shown in Fig. 2C and D in which fragment b detected in the 2+22 h samples disappeared in the 2+34 h samples. Importantly, the fragment b did not disappear in the 2+34 h sample incubated in the presence of ac-VEID-CHO, a caspase-6-specific inhibitor (Fig. 3B), further supporting the caspase-6-mediated fragmentation of fragment b in Aβ-treated cells.

The fragment a could be produced by caspase-6-mediated fragmentation of fragment b in the cell and cell-free experiments (see Fig. 2G). Although it was not detected in the 90 min-incubated ANU sample (Fig. 3A), a faint band corresponding to the fragment was in an ANU sample incubated for 60 min with caspase-6 (Fig. 3C). We speculated that these results reflect the instability of fragment a.

Based on these observations and our previous report showing that NSP-mediated generation of lamin A fragment preceded the caspase-6 activation [4], we suggest that Aβ induces NSP-mediated fragmentation of lamin A protein to produce fragment b which is further fragmented by caspase-6 (summarized in Fig. 3D).

### 3.4 Probing the fragmentation of lamin B by caspase-6 in isolated nuclei and cells

**Fig. 4:**
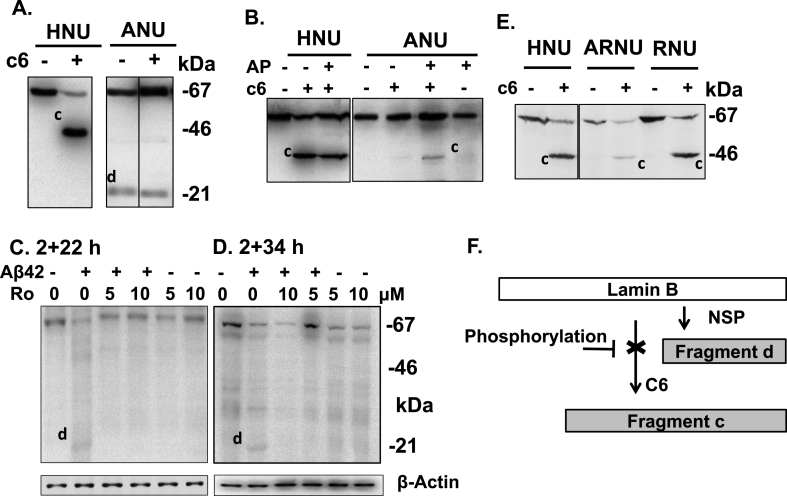
Fragmentation of lamin B by caspase-6. (A, B and E) HNU and ANU were prepared as indicated in Fig. 3. ARNU is nuclei isolated from cells pre-incubated for 2 h with 10 μM of roscovitine and then treated with 10 μM of Aβ42 for 2 h with roscovitine followed by another incubation with 10 μM of Aβ42 and roscovitine for 22 h, while RNU is nuclei isolated from cells treated as ARNU cells without Aβ42. Nuclei were incubated without and with 50 ng of purified recombinant caspase-6 for 1.5 h at 30 °C. 4 U of AP was also included in the reaction mixture in B. (C and D) Cells were treated as indicated in Fig. 2D with and without roscovitine. Fragmentation of lamin B was determined by western blot analysis in all experiments. β-Actin was used as a control in C and D. Relative molecular weights (in kDa) are indicated on the right. c, d, c6 and Ro are fragment c, d, caspase-6 and roscovitine, respectively. Data are representative of 3 independent experiments. (F) Schematic drawing of lamin B fragmentation process.

In HNU lamin fragments corresponding to fragments c were generated for lamin B by caspase-6 (Fig. 4A, HNU panel) as expected in the western blot analysis. However, the fragment was not detected in ANU treated with caspase-6 (Fig. 4A, ANU panel), implying that the lamin B protein of ANU was resistant to the proteolytic activity of the caspase due to an unknown reason. This result is consistent with those of Fig. 2C and D showing the absence of fragment c in Aβ-treated cells albeit activation of caspase-6. We hypothesized that a modification such as phosphorylation of lamin B by protein kinases [18] could inhibit the caspase-6-mediated fragmentation of the protein. Thus, we explored whether caspase-6 produces lamin fragment c in ANU when a phosphatase (AP, here) is included in the reaction mixture. While AP showed little effect on the production of lamin B fragments by caspase-6 in HNU, ~46 kDa fragments of lamin B corresponding to fragment c were newly detected in the AP-containing samples (ANU panel of Fig. 4B). Thus, we propose that phosphorylation is at least in part responsible for prevention of lamin B fragmentation by caspase-6 in Aβ-treated cells.

Lamin proteins can be phosphorylated by several kinases. For example, the phosphorylation of lamin B at S395 and S405 sites by protein kinase C leads to nuclear degradation and DNA fragmentation in apoptotic HL60 cells [19,20]. Other examples include Cdc2 kinase and Akt that can phosphorylate lamin B during mitosis [21] or in response to insulin [22]. These kinases, however, contribute to nuclear irregularities in proliferating cells [[19], [20], [21], [22]]. On the other hand, Aβ peptide-induced phosphorylation was reported to be directly correlated with Cdk5 that mediated breakdown of the nuclear envelope in Aβ-treated cells and phosphorylation at S22 and S392 in lamin A and S23 and S393 in lamin B1 [18]. Thus, we examined the fragmentation of lamin B in cells treated with Aβ42 in the presence of roscovitine, a Cdk5 inhibitor, to explore the involvement of the kinase in the inhibitory phosphorylation on lamin B protein.

However, the initial analysis of cells treated with Aβ42 in the presence of roscovitine for lamin B fragmentation was not successful, because this experimental condition resulted in more cell death (data not shown) and thus the western blot analysis of those cells could be misleading (Fig. 4C and D, in which the fragment c was not detected in the cells). Thus, instead of the investigation of cells treated as above, we tested the fragmentation of lamin B in cell-free experiments wherein nuclei isolated from cells treated with Aβ42 in the presence of roscovitine (ARNU) were incubated with purified caspase-6. Fragment c was detected in ARNU treated with caspase-6 (ARNU panel, Fig. 4E), indicating that inhibition of Cdk5 led to lamin B protein becoming susceptible to the caspase and strongly supporting the involvement of the kinase in the inhibitory phosphorylation on lamin B protein.

Based on the current study, a schematic diagram regarding fragmentation of lamin B protein in Aβ-treated cells was provided in Fig. 4F. In short, lamin B is fragmented to fragment d in Aβ-treated cells, but caspase-6-mediated cleavage of lamin B did not occur in the cells, probably due to phosphorylation of the protein by Cdk5.

## 4 Discussion

**Fig. 5:**
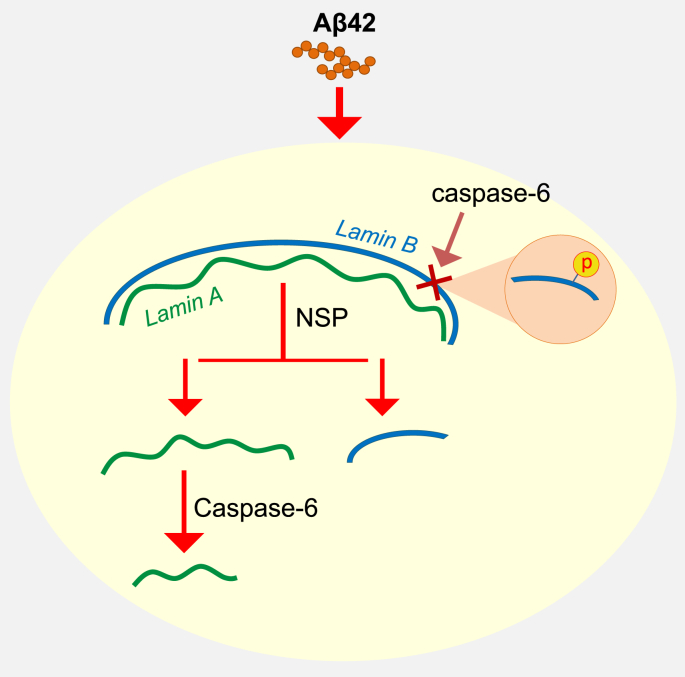
Graphical abstract of lamin A and B fragmentation process. Aβ42 induces NSP-mediated proteolytic fragmentation of lamin A and B. The NSP cleaved products of lamin A are further degraded by caspase-6. However, phosphorylation of lamin B makes its inaccessible to caspase-6 under Aβ toxicity.

A graphic abstract of the current study is presented in Fig. 5. One novel observation in this study was the role of caspase-6 in removal of fragment b generated by NSP-mediated fragmentation of lamin A in Aβ42-treated cells (Fig. 3). The removal pathway is a subsequent process after the initial fragmentation of lamin A by NSP. Thus, we speculate that the NSP-mediated lamin fragmentation is an early event leading to the apoptotic process in the death process induced by Aβ peptide.

The other new observation here was a possible involvement of phosphorylation of lamin B in inhibition of the protein fragmentation by caspase-6 (Fig. 4). How phosphorylation can affect the fragmentation of lamin B is yet to be determined. The phosphorylation sites of Cdk5 (S23 and S393 of lamin B1) are far from the caspase-6 cleaving sites [C-terminus of D230 of lamin A or D231 of lamin B (see Fig. 2G)]. Thus, the inhibitory effect of phosphorylation on the cleavage of lamin proteins by caspase-6 seems to be mediated in an indirect manner. Possibly, a structural change in the lamin proteins by phosphorylation might have the inhibitory effect.

The previous observation showing non-apoptotic lamin fragmentation in the death process induced by Aβ peptide [4] was serendipitous, because caspase-6, implicated in neurodegenerative disease such as AD [23], is a well-known enzyme catalyzing the fragmentation of lamin proteins in processes induced by several different types of damaging agents [4]. The current observations are compatible with the previous report [4] in that lamin fragmentation is mediated by NSP and largely independent of caspase-6 activation in Aβ-treated cells. As far as we know, the pathway leading to lamin protein fragmentation shown here is specific for Aβ. NSP appears to be a key player in the pathway leading to lamin fragmentation in Aβ-treated cells. Thus, one of questions that should be answered in the following study is the recognition of identity of NSP responsible for the fragmentation of lamin proteins. We are currently pursuing a research project for it.

Lamina dispersion and deformation are important in the process of neuronal cell death [24,25]. And we previously showed that the lamin fragmentation was well correlated with Aβ toxicity [4]. Thus, current and following studies to understand the fragmentation process will provide novel insights into Aβ-associated AD pathology and hopefully a new therapeutic target to control the disease.

## Author contributions

**Il-Seon Park (Corresponding Author/Chief Investigatior)**: project administration, writing and editing, supervision.

**Md. Imamul Islam and Md. Selim Hossain:** Methodology, investigation, resources.

## Declaration of competing interest

The authors declare that they have no known competing financial interests or personal relationships that could have appeared to influence the work reported in this paper.
